# Research progress on antiviral drugs and vaccines for severe fever with thrombocytopenia syndrome

**DOI:** 10.3389/fimmu.2026.1730089

**Published:** 2026-01-27

**Authors:** Chendan Chen, Jianhua Li, Jiaxuan Li, Renjin Huang, Chenghao Chen, Jinghan Xu, Yanjun Zhang, Yongliang Lou

**Affiliations:** 1 Zhejiang Key Laboratory of Public Health Detection and Pathogenesis Research, Department of Microbiology, Zhejiang Provincial Center for Disease Control Prevention, Hangzhou, China; 2 School of Laboratory Medicine and Life Sciences, Wenzhou Medical University, Wenzhou, China; 3 School of Basic Medical Sciences, Zhejiang Chinese Medical University, Hangzhou, China; 4 School of Medical Technology and Information Engineering, Zhejiang Chinese Medical University, Hangzhou, China

**Keywords:** antiviral drugs, drug therapy, SFTS, SFTSV, vaccine

## Abstract

Severe fever with thrombocytopenia syndrome (SFTS), caused by the SFTS virus (SFTSV), has emerged as a significant global public health threat. Infected patients may present with gastrointestinal, neurological, and cardiovascular ribavirin and favipiravir are currently used in clinical practice, their efficacy remains controversial, and treatment primarily relies on symptomatic and supportive care. To date, there is no standard treatment regimen for SFTSV infection, nor are there any approved vaccines. However, recent advances in SFTSV research and the application of novel technologies have opened new pathways for the development of antiviral drugs and vaccines. This review summarizes the latest progress in the development of therapeutic agents and vaccines against SFTSV, aiming to provide valuable insights for drug development and countermeasure strategies for SFTS.

## 1 Introduction

Severe Fever with Thrombocytopenia Syndrome (SFTS) is an acute infectious disease caused by the Severe Fever with Thrombocytopenia Syndrome Virus (SFTSV). SFTSV is also known as the Dabie bandavirus, Dabie Mountain Virus, or Huaiyangshan Virus. Since 2022, the International Committee on Taxonomy of Viruses (ICTV) has named it ‘Bandavirus dabieensee’ (1). To maintain consistency in the narrative, this article continues to use the term SFTSV. SFTSV was first isolated in China in 2009 (2), and subsequent confirmed cases have been reported in East Asia (e.g., South Korea, Japan) and Southeast Asia (e.g., Vietnam, Pakistan) (3–7). The United States has reported a virus similar to SFTSV, known as the Heartland Virus (HRTV) (8). In 2018, a novel tick-borne pathogen named Guertu virus (GTV) was reported, which was first isolated from *Dermacentor nuttalli* ticks collected in 2014 in the Xinjiang Uygur Autonomous Region of China. GTV is closely related to SFTSV and HRTV (9).

The critical period of the disease course is 7-13 days after SFTSV infection. Persistent high serum viral load indicates a high risk of disease deterioration or death (10). Almost all patients experience fever (≥38°C), often accompanied by gastrointestinal symptoms such as nausea and vomiting. Some patients also exhibit neurological symptoms such as fatigue, myalgia, and altered consciousness (11). Laboratory abnormalities commonly include thrombocytopenia, leukopenia, significantly elevated alanine aminotransferase (ALT) and aspartate aminotransferase (AST) levels, and proteinuria/hematuria, indicating abnormal liver and kidney function (12). Nearly half of patients exhibit coagulation disorders and elevated cardiac injury markers. Severe cases may progress to coma, bleeding, multiple organ failure (13), or even death. In 2017, the World Health Organization (WHO) designated SFTS as a priority disease (14). The case fatality rate of Severe Fever with Thrombocytopenia Syndrome (SFTS) exhibits considerable variation across different countries. According to data from Japan’s National Vital Statistics Surveillance System (NVSSS), the SFTS case fatality rate in Japan remained consistently high, averaging around 20% from 2013 to 2022 (15). In contrast, reports indicate that as of 2023, South Korea’s SFTS case fatality rate was approximately 18.7% (16), while China reported a comparatively lower rate of approximately 4.82% (17).Meta-analysis shows that the mortality rate is higher in males than in females (18), and the mortality rate increases significantly with age, reaching 17.40% in the group aged 80 years and above. The period from July to September each year has the highest mortality rate (18).

The virus is predominantly transmitted by ticks, with *Haemaphysalis longicornis* identified as the principal vector (19). In addition, various domestic animals, including cattle, goats, sheep, pigs, cats, and dogs, serve as significant carriers (20), thereby substantially elevating the risk of SFTS outbreaks. Research indicates that the primary transmission hosts vary across different regions in China, with poultry, such as chickens, ducks, and geese, frequently identified as high-contribution hosts (21). Beyond animal vectors, SFTSV can also be transmitted via human-to-human contact, primarily through exposure to patients’ blood or blood-containing secretions during cluster outbreaks (22, 23). Activities such as patient care, funeral rituals, and medical procedures all carry a high risk of infection (24). The ongoing expansion of the geographical range of transmission vectors, combined with the high case-fatality rate and severe multi-organ failure linked to SFTS, constitutes a significant global public health threat. Presently, there is no standardized treatment protocol for SFTSV, nor is there a commercially available vaccine. Patients primarily rely on symptomatic and supportive care (25). While broad-spectrum antiviral drugs ribavirin and favipiravir have demonstrated efficacy *in vitro* studies, their clinical trial outcomes have not met expectations due to limited efficacy and adverse side effects. The development of other antiviral agents is often hindered by unclear antiviral mechanisms, high research and development costs, routes of administration, and the absence of pharmacokinetic and toxicological evaluations. Most remain stagnant at the *in vitro* or animal trial stage and require further research and optimization, having not yet entered clinical validation. Therefore, the development of effective SFTSV antiviral drugs is urgently needed.

## 2 Characteristics and pathogenic mechanisms of SFTSV

### 2.1 Characteristics of SFTSV

SFTSV is classified within order *Bunyavirales*, family *Phenuiviridae*, and genus *Bandavirus*. The viral particles exhibit a spherical icosahedral structure with a diameter of approximately 80–120 nm and are enveloped in a lipid bilayer membrane derived from the host cell membrane. Cryo-electron microscopy has revealed that the surface of SFTSV is covered with penton and hexon spikes, containing a total of 720 Gn-Gc heterodimers (26). SFTSV is a single-stranded, negative-strand RNA virus. Its genome consists of three fragments: small, medium, and large, containing 1,744, 3,378, and 6,368 nucleotides, respectively (2). Segment L encodes RNA-dependent RNA polymerase (RdRp) required for viral replication and transcription. Structural analysis of the L protein, covering approximately 70% of its sequence, reveals a conserved RdRp domain, an endonuclease domain connected by a flexible linker region, and a cap-binding domain (CBD). Functional assays have demonstrated that the L protein must concurrently bind to the 3’ and 5’ promoter RNAs of the viral genome to activate its Mg^2+^/Mn^2+^-dependent polymerase, facilitating replication through terminal initiation (27). The medium segment (M) encodes the precursors of glycoproteins Gn and Gc, which are cleaved into two glycoproteins, Gn and Gc, that play crucial roles in viral particle assembly and entry into host cells. The small segment (S) encodes the non-structural protein (NS) in the forward direction and the nucleoprotein (NP) in the reverse direction, with a 62-base pair spacer region between them. The NP forms a hexamer and interacts with viral RNA to form viral ribonucleoprotein (RNP) (2).

### 2.2 SFTSV genotyping and potential clinical correlations

SFTSV demonstrates considerable genetic diversity, with multiple genotypes primarily identified through whole-genome sequencing and phylogenetic analysis. Despite this, a universally accepted genomic typing and nomenclature system has not yet been established. Initial research categorized SFTSV into five genotypes, labeled A-E (28, 29). Subsequent investigations refined this classification to six genotypes, A-F (30–32), which has become the more commonly used classification method. Alternative classification frameworks have also been proposed, including a system that organizes the virus into three lineages, I-III, with lineage I further divided into two sublineages (33), and another that divides the virus into two clades, C and J, forming eight genotypes: C1-C5 and J1-J3 (34). Additionally, another study identified seven distinct branches, comprising five Chinese lineages (I, II, III, IV, and V) and two Japanese lineages (VI and VII) (35).

There is evidence suggesting potential correlations between specific genotypes and clinical pathogenicity (36). For instance, analysis of case fatality rates among different genotypes reveals that the B2 genotype is associated with higher mortality (37), which may partially account for the increased death rates observed in South Korea and Japan, where B-type epidemics are prevalent.

Association analyses between clinical indicators and genotypes have demonstrated that certain genotypes linked to increased mortality rates may provoke more robust inflammatory responses (35, 38). Research indicates that patients classified within the Clade IV branch are at a heightened risk of mortality, exhibiting significantly elevated levels of inflammatory mediators compared to other branches. A distinctive co-mutation pattern within this branch is associated with an increased risk of mortality and pronounced inflammatory responses (35). At the molecular level, variations in the M gene fragment are posited to play a pivotal role in the observed genotypic differences (38–40). These mutations are likely intricately linked to the virus’s mechanisms of immune evasion and host adaptation. For example, the V506M mutation in the Gn glycoprotein region of the M fragment from the A genotype strain in Henan Province, China, has been implicated in adverse clinical outcomes (41). Additionally, a study focusing on various subclones of the SFTSV strain YG1 identified the R624W mutation in the Gc protein as a critical site influencing the virus’s low pH-dependent cell fusion (42). The evolutionary rates of SFTSV’s three genomic segments (L, M, and S) also vary, with the S segment evolving most rapidly and exhibiting the strongest adaptive capacity (43). Overall, SFTSV shows relatively conserved evolution. However, due to factors such as tick vectors, climate change, and human agricultural activities, enhanced surveillance remains essential.

### 2.3 Pathogenic mechanism

#### 2.3.1 Viral target cells

Initial investigations indicated that monocytes/macrophages are the main target cells of SFTSV (44, 45). The infection prompts monocytes to transition into the CD14^+^CD16^+^ intermediate subtype (46, 47), reduces the numbers of T cells, dendritic cells, and NK cells, promotes M2 macrophage differentiation, and triggers host immune dysregulation. In-depth studies have revealed that macrophages are not the main target cells in non-lymphoid organs, and their role is more aligned with immune regulation rather than acting as vectors for viral transmission vectors (48). The central mechanism underlying lethal infection involves impaired B-cell class switching and the generation of nonfunctional plasmablasts, leading to a deficiency in specific IgG, which culminates in sustained high viremia and systemic immune dysregulation (48–50). The failure of the humoral immune response is attributed to a progressive immune dysfunction. Early apoptosis of monocytes compromises the differentiation and function of myeloid dendritic cells, which in turn leads to follicular helper T cell dysfunction. This cascade ultimately results in disrupted B-cell immunity, maturation defects, and proliferation of plasmablasts with a concomitant loss of antibody secretion capability (49). Additionally, fibroblastic reticular cells in intestinal and splenic lymphoid tissues are also important targets (51).

#### 2.3.2 Invasion pathways

SFTSV invasion of host cells is a complex biological process that mainly includes attachment, endocytosis, endosomal transport, and acidification. The virus can enter dendritic cells and specific cell lines via the dendritic cell-specific intercellular adhesion molecule-3-grabbing non-integrin (DC-SIGN) (52). It can also utilize the chemokine receptor C-C Motif Chemokine Receptor 2 (CCR2), which is expressed on monocytes, macrophages, and plasma cells, and whose N-terminal extracellular domain can directly bind to the viral Gn protein (53). The CLI and CLII domains of prolow-density lipoprotein receptor-related protein 1 (LRP1) can also directly bind to Gn protein (54), and together with the DC-SIGN receptor and CCR2, they mediate the initial anchoring of the virus. Non-muscle myosin heavy chain IIA (NMMHC-IIA), which is widely present in susceptible cells such as endothelial cells and megakaryocytes, can specifically bind to the Gn protein to participate in the viral entry process, and its functional loss significantly inhibits infection (55). In addition, the virus can invade through a Talin1-dependent lipid raft endocytosis pathway (56). Platelet-derived growth factor receptor β (PDGFRβ) has also been demonstrated to participate in the entry process of SFTSV (57). Notably, glucosylceramide synthase (UGCG) influences the post-endocytic stage of SFTSV entry by regulating the initiation of glycosphingolipid synthesis (58). The host factor sorting nexin 11 (SNX11) promotes viral penetration from endolysosomes into the cytoplasm by maintaining endosomal acidification and late endosomal homeostasis, serving as a crucial host factor in viral invasion (59).

#### 2.3.3 Mechanisms of thrombocytopenia

Thrombocytopenia is a common symptom in SFTSV patients (2). Initial studies identified the phagocytosis of SFTSV-platelet complexes by splenic macrophages as the primary cause. Based on the viral load observed in platelets during virus culture and the unchanged viral load in the supernatant, it was inferred that the virus did not replicate within platelets (60). However, new evidence suggests that SFTSV can utilize the translational machinery of platelets to synthesize its own proteins, hijack the autophagy pathway, and recruit the ER-Golgi intermediate compartment (ERGIC) and Golgi apparatus to form viral factories for viral particle assembly and release, thereby promoting its own replication. This process is characterized by delayed release, and previous studies may have overlooked the significant increase in viral release during the later stages due to shorter co-culture times (61). The platelet membrane GPVI receptor is one of the critical binding receptors for SFTSV. On one hand, platelets mediate a protective response by inhibiting macrophages from producing inflammatory cytokines, such as IL-6 and TNF-α, thereby alleviating the cytokine storm. On the other hand, platelets serve as efficient carriers, significantly enhancing macrophage phagocytosis of SFTSV viral particles. Viruses phagocytosed by macrophages replicate within the cells, producing a large number of progeny viruses, which are then released into the bloodstream after inducing cell pyroptosis (61, 62). This leads to increased platelet consumption, forming a vicious cycle of “phagocytosis-replication- apoptosis-release-reinfection.” This cycle is considered one of the key mechanisms underlying the persistent platelet depletion observed in SFTS. Notably, SFTSV exhibits significantly higher replication capacity in activated platelets compared to resting platelets; however, the underlying mechanisms remain unclear and require further investigation (61).

Additionally, the virus-induced abnormal platelet function and increased platelet death are also mechanisms contributing to thrombocytopenia. Transcriptomic analyses of platelets from patients with SFTS have revealed functional disorders characterized by neutrophil activation and neutrophil extracellular trap (NET) formation, activation of the interferon signaling pathway, and dysregulation of cytokine and chemokine expression. These abnormalities collectively drive excessive platelet consumption. Furthermore, platelets in patients with SFTS are disrupted by multiple apoptotic mechanisms. Increased pyroptosis may stimulate inflammasomes and release cytokines, thereby enhancing platelet aggregation, increasing endothelial permeability, and exacerbating inflammatory responses, further accelerating platelet depletion (63).

#### 2.3.4 Host immune dysregulation

SFTSV mediates systemic immune evasion and cytokine storms through its principal virulence proteins, NSs and NP. NSs, as a core virulence factor, systematically disrupts the host’s type I interferon pathway by hijacking the autophagy mechanism via inclusion bodies. It sequesters TRIM25 (64) and binds to LSm14A (65) to inhibit RIG-I signaling, while also sequestering the TBK1/IKKϵ/IRF3 (66, 67) complex and IRF7 (68) to impede interferon synthesis. Additionally, it intercepts STAT1/STAT2 to obstruct JAK-STAT signaling and silence ISG expression (69–71). The virus further aberrantly activates pro-inflammatory signals, leading to excessive activation of the IKKβ-NF-κB axis through inhibition of TBK1, which triggers a cytokine storm (72, 73). At the same time, it utilizes the TPL2-IL-10 axis to mediate immune suppression and promote self-replication (74). The mechanism of its inflammatory spread includes phase separation assembly of the ‘NSs-RIPK3-MLKL necrosome,’ which activates RIPK3-dependent necroptosis (75). Moreover, it interacts with NLRP1/CARD8 and induces degradation of DPP8/9 (dipeptidyl peptidases 8 and 9), thereby activating the inflammasome to trigger GSDMD pyroptosis and IL-1β release (76). Furthermore, the virus inhibits the formation and nuclear translocation of cyclin B1-CDK1 by sequestering CDK1, inducing G_2_/M phase arrest in cells to facilitate viral replication (77).

SFTSV NP induces interferon production and inflammation by interacting with scaffold attachment factor A (SAFA) to retain it in the cytoplasm and activating the SAFA-STING-TBK1 signaling pathway (78). Conversely, viral NSs proteins inhibit SAFA-mediated innate immune responses and promote viral replication by mediating the autophagic degradation of SAFA via LC3/SQSTM1 (79). By interacting with Tu translation elongation factor, mitochondrial (TUFM), the SFTSV NP translocates to the mitochondria, where it facilitates the degradation of mitochondrial antiviral signaling protein (MAVS) via LC3-mediated mitochondrial autophagy in order to inhibit antiviral immunity (80). Infection- induced BCL2 antagonist/killer 1 (BAK) and BAK/BCL2-associated X (BAX) mediate mitochondrial membrane permeabilization, resulting in the release oxidized Mitochondrial DNA (mtDNA) (84). On the one hand, the cGAS-STING pathway is activated, but NP-mediated binding of Cyclic GMP-AMP synthase (cGAS) to LC3 and sequesters it into autophagosomes for degradation, thereby antagonizing this pathway (81). On the other hand, oxidized mtDNA activates NLRP3 inflammasomes, inducing caspase-1 activation, which in turn promotes the maturation and release of IL-1β (82, 83). The BAK/BAX-mtDNA-NLRP3 axis plays a pivotal role in determining disease severity, with BAK levels serving as a potential prognostic marker (84). Furthermore, the N-terminal fragment of NSs facilitates the assembly of the NLRP3-ASC-caspase-1 complex by interacting with NLRP3, which subsequently leads to the maturation and release of IL-1β and induces cellular pyroptosis (85).

The envelope glycoprotein Gn impedes the NF-κB and IRF3 signaling pathways by binding to and degrading STING, thereby diminishing the immune response (86). Additionally, the virus exploits the host p38 protein and induces phosphorylation of p38 to activate the MAPK signaling pathway, thereby promoting its own replication (87). The virus also triggers the activation of the CyPA-CD147-MAPK pathway, which induces the release of inflammatory factors and aggravates tissue damage (88).

In conclusion, the pathogenic mechanisms of SFTSV encompass synergistic multi-receptor endocytic invasion, NSs/N protein-mediated interferon suppression and inflammatory storm, disruption of humoral immunity due to the expansion of non-functional plasmablasts, thrombocytopenia, and coagulation dysfunction. Ultimately, the host succumbs to endothelial damage, T-cell depletion, and multi-organ failure. Transcriptome analysis revealed that genes exhibiting downregulation, such as *GP1BA and FLNA*, were predominantly associated with platelet activation and coagulation pathways, aligning with the clinical manifestation of thrombocytopenia. Genes that were upregulated, including IFITM1 and IFITM3, were primarily involved in antiviral immune responses and inflammatory pathways. At the epigenetic level, elevated expression of the demethylase FTO may facilitate viral replication through m^6^A demethylation (89). The virus further exploits m^6^A regulators through its NP to regulate m^6^A modification of viral RNA. This modification enhances the translational efficiency of SFTSV NP and the stability of viral RNAs, and significantly facilitates viral infection (90). This elucidated mechanism provides a theoretical foundation for the development of targeted inhibitors. In addition, the integration of omics technologies, including genomics, transcriptomics, proteomics, and metabolomics, alongside high-resolution real-time dynamic imaging, gene editing, and other techniques, can be further utilized to deepen the understanding of the mechanism, thereby offering a more robust theoretical basis for precision drug design.

## 3 Current status of research on existing anti-SFTSV drugs

### 3.1 Enzyme inhibitors

#### 3.1.1 Ribavirin

Ribavirin exhibits broad-spectrum antiviral properties, demonstrating efficacy against pathogens such as Crimean-Congo hemorrhagic fever virus and Lassa virus (91–93). It can be administered via orally, intravenously, or nebulized inhalation (94). The drug’s mechanism of action involves inhibiting viral RdRp activity, inducing viral genome mutagenesis, impeding the RNA capping process, decreasing cellular inosine monophosphate dehydrogenase activity, and modulating the host immune response (95). *In vitro* studies have confirmed its antiviral efficacy (96–98). However, some animal studies (99, 100) and clinical studies (101–103) have shown that its efficacy is controversial. It is worth noting that clinical practice suggests that the combination of ribavirin and plasma exchange may have certain efficacy (104, 105).

First, the efficacy of ribavirin is closely linked to early administration, with preemptive use prior to significant viral load escalation being crucial (106, 107). A study conducted in China on SFTS indicated that ribavirin was effective only in patients with low viral loads (<1×10^6^ copies/mL) and showed no significant benefit in those with high viral loads (≥1×10^6^ copies/mL) (108). *In vitro* experiments further demonstrated that adding ribavirin before viral infection effectively inhibited the virus, but adding it three days after infection significantly reduced its efficacy (96). A clinical observation conducted in Hefei, China, demonstrated that the mortality rate was lower in patients treated within five days of onset compared to the group receiving late treatment. However, the difference in mortality rates between the untreated and treated groups did not achieve statistical significance in this study (109). This result is consistent with other studies suggesting that ribavirin has limited efficacy in reducing mortality (101, 103, 110).

Secondly, the therapeutic effect of ribavirin may be selective for patient subsets and requires stratification modeling based on patient criticality. A composite score incorporating age, SFTSV RNA load, and gastrointestinal bleeding was constructed, and patients were grouped according to neurological symptoms. The results indicated that ribavirin was effective only in the ‘single-positive group’ (patients with positive indicated scores or neurological symptoms) (110).

Thirdly, ribavirin treatment may trigger side effects, mainly in the form of a significant decrease in hemoglobin levels and an increase in blood amylase. Anemia and hyperamylasemia may occur in SFTS patients treated with this drug (111).

#### 3.1.2 Favipiravir

Favipiravir (T-705) is a broad-spectrum viral RNA polymerase inhibitor initially approved in Japan for the treatment of novel or emerging influenza virus infections. Based on data from its SFTS clinical trial, the medicine has been submitted for an expanded indication (112). Data suggest that favipiravir could decrease the mortality rate of SFTS patients in Japan by approximately 10% (113).

T-705 can be converted to phosphoribosylated metabolites (114). It induces base mismatches, mainly through the inhibition of RdRP function. *In vivo*, these mismatches manifest as both transition and transversion mutations, many of which are deleterious and non-synonymous. Under T-705 pressure, relatively higher frequencies of mutations accumulated in the L and M genomic fragments (99), leading to the accumulation of mutations and error catastrophe of the viral genome, and thus inhibition of viral replication (99, 115–117).

This drug exhibits significant inhibitory activity against viruses such as Ebola and Lassa virus, with potentially stronger effects against SFTSV (118, 119). T-705 may demonstrate superior efficacy compared to ribavirin (99, 120), and its anti-SFTSV activity has been validated through *in vitro* experiments (98, 99, 121, 122), animal models (99, 119–122), and clinical practice (123).

The efficacy of T-705 exhibits clear dose dependency and time sensitivity. Higher dosages of T-705 demonstrate enhanced antiviral effects in both cellular and animal models (99, 120). *In vitro* studies demonstrate that the concentration of T-705 necessary to inhibit SFTSV is greater than that required to inhibit the influenza virus. Specifically, the IC_50_ for influenza virus inhibition ranges from 0.013–0.48 μg/mL (124), while the EC_50_ for SFTSV inhibition is 4.14 µg/mL, which is also higher compared to other analogous antiviral agents (125). This disparity may constrain the clinical utility of T-705.

Furthermore, animal studies indicate that T-705 should be administered as early as possible post-infection (119, 121). This suggests its efficacy may be limited in patients with high viral loads or those who have progressed to severe disease. Clinical research shows that patients with viral loads ≥ 1 × 10^5^ copies/mL exhibit a mortality rate of 40%, which is significantly higher than that observed in patients with low viral loads (113).

This drug demonstrates an overall favorable safety profile, with serious side effects being rare and drug resistance infrequent (99, 121). However, some patients treated with favipiravir may experience symptoms such as abnormal liver function (113, 126, 127), red itchy rashes (126), vomiting, nausea, diarrhea, and hyperuricemia (127). Whether these symptoms constitute side effects of favipiravir treatment remains to be confirmed. Regarding administration, oral and intravenous routes demonstrate comparable antiviral activity (121). However, intravenous administration is recommended for patients with severe central nervous system or gastrointestinal symptoms (113). Furthermore, caution is recommended when administering this medication to this demographic, considering the possibly restricted therapeutic benefit in patients aged 70 years or older (127).

### 3.2 Calcium channel blockers

Calcium channel blockers (CCBs), besides their extensive application in cardiovascular and cerebrovascular disorders, have been found to have antiviral activity against a number of lethal viruses, including hantaviruses (128), Ebola viruses (129), Marburg viruses (130), and SFTSV. *In vitro* studies indicate that CCBs such as benidipine hydrochloride and nifedipine exhibit dose-dependent inhibitory effects against SFTSV, primarily through the inhibition of calcium ion influx and reduction of intracellular Ca^2+^ concentration. Calcium-free medium, calcium chelator (BAPTA-AM), or knockdown of the L-type calcium channel Cav1.2 gene inhibited viral replication, confirming that calcium channels are a key target (131). A clinical case report of a 67-year-old male patient who failed steroid therapy and progressed to hemophagocytic lymphohistiocytosis (HLH) recovered completely and without complications after receiving supportive care combined with intravenous nicardipine for hypertension, suggesting that the drug may be beneficial for his recovery (132). Loperamide (133) and manidipine (134) exert their anti-SFTSV effects by inhibiting the replication phase of the virus after entry into the host cell. Among them, manidipine is effective *in vitro* and *in vivo*, with broad-spectrum activity against a wide range of negative-stranded RNA viruses and the capacity to inhibit inclusion body formation induced by viral nucleocapsid proteins (134). Mechanistic studies have shown that calcium ions play an important role in viral replication, influencing both endocytosis and the regulation of calcium-modulated calcineurin-NFAT pathway and actin dynamic equilibrium (134).

### 3.3 Nucleoside analogues

#### 3.3.1 2’-Fluoro-2’-deoxycytidine

2’-Fluoro-2’-deoxycytidine (2’-FdC) demonstrates antiviral efficacy against a range of viruses *in vitro*, including Lassa virus, Crimean-Congo hemorrhagic fever virus, and Rift Valley fever virus. Administration of 2’-FdC via intraperitoneal injection resulted in a significant reduction in mortality, with the 100 mg/kg/day cohort achieving 100% survival. However, all dosage groups exhibited notable weight loss, whereas the favipiravir group (100 mg/kg/day) maintained stable body weight with 90% survival. By day 5 post-infection, only the 100 mg/kg 2’-FdC group exhibited a significant decrease in serum and tissue viral loads, with no statistically significant differences observed in the other dosage groups. The administration of a higher dose (200 mg/kg) of 2’-FdC did not enhance efficacy and may suggest a potential toxic effect, necessitating further investigation. 2’-FdC was ineffective in the La Crosse virus encephalitis model, suggesting its limited therapeutic effect against neuroinvasive infections, presumably due to its inability to effectively cross the blood-brain barrier (135).

#### 3.3.2 4′-Fluorouridine and its double prodrug VV261

The nucleoside antiviral drug 4′-Fluorouridine (4-FU) efficiently inhibits SFTSV replication *in vitro*, with significantly superior efficacy compared to T-705. It did not elicit significant adverse reactions in animal trials, suggesting its promise as a viable therapeutic approach against SFTSV and other bunyaviruses (136). The novel modified prodrug VV261 exhibits significantly enhanced chemical stability and favorable pharmacokinetic properties. In a fatal SFTSV infection scenario, sustained dosing of VV261 for 7 days provided complete protection. Treatment for only two days significantly reduced viral load and infectious viral titers in multiple organs while markedly alleviating splenic pathological damage, establishing it as a breakthrough candidate drug for SFTS treatment. Currently, VV261 has entered Phase I clinical trials in China as the first-in-class drug. However, its inadequate water solubility may restrict high-dose oral absorption, and interspecies pharmacokinetic differences could impact initial human dose estimation (137).

#### 3.3.3 Fludarabine

Fludarabine is a nucleoside analog prodrug optimized from vidarabine, which is metabolized into F-ara-A *in vivo* to exert its therapeutic effect. Its efficacy is mainly attributed to the multiple inhibition of DNA synthesis, with some studies demonstrating its ability to inhibit RNA synthesis as well (138). Notably, Fludarabine effectively inhibits infection by various RNA viruses, including Zika virus, SFTSV, and enterovirus A71. The half-maximal inhibitory concentration (IC_50_) values in Vero, BHK-21, U251 MG, and HMC3 cell lines were all below 1 μM (139). Nonetheless, its potential for translation into an SFTS therapeutic is limited by issues including its cytotoxicity, unclear mechanism of action, narrow therapeutic window, lack of *in vivo* validation data, and known clinical toxicity (139).

### 3.4 Other antiviral drugs

#### 3.4.1 Caffeic acid

Upon mixing caffeic acid (CA) with SFTSV in a culture medium and subsequently inoculating it into cells, CA was observed to specifically inhibit viral infection in a dose-dependent manner (IC_50_ = 0.048 mM, SI = 158), and its effect was independent of acidity. Pre-incubation enhanced the CA inhibitory effect (140). Conversely, the addition of CA post-infection significantly diminished its antiviral activity. Mechanistic investigations indicate that CA primarily blocks viral binding to host cells by disrupting viral surface structures, without interfering with replication of viruses already inside cells. These findings suggest CA may be suitable only for prophylactic intervention before substantial viral replication occurs. Its therapeutic value for infected individuals with already existing clinical symptoms may be limited, constraining its potential for clinical application (140).

Analysis of the structure-activity relationship indicates that the o-dihydroxybenzene framework is essential for antiviral activity. Compounds containing this structure (such as 3,4-dihydroxyhydrocinnamic acid (DHCA), catechol, and their CA derivatives) all exhibit dose-dependent antiviral activity. However, this skeleton is also the primary source of cytotoxicity (141). Therefore, it is recommended to modify the o-dihydroxybenzene skeleton chemically to develop novel drugs with higher efficacy against SFTSV and lower toxicity. Additionally, 3-Hydroxy-L-tyrosine (L-DOPA), which possesses this skeleton, also exhibits antiviral efficacy. Its synthetic enantiomer, 3-hydroxy-D-tyrosine (D-DOPA), theoretically may carry a lower risk of side effects (142). L-DOPA is efficiently metabolized *in vivo* by dopa decarboxylase (DDC) and catechol-O-methyltransferase (COMT). The combination of metabolic enzyme inhibitors extends the *in vivo* residence time of L-DOPA and yields synergistic effects. Among these, COMT inhibitors (e.g., entacapone) exert efficacy during the post-infection treatment phase (143). Similarly, green tea polyphenols (e.g., epigallocatechin gallate) with a trihydroxybenzene structure exhibit stronger CA activity than dihydroxybenzene-containing compounds, but they primarily block viral adsorption through pretreatment as well (144). Currently, research on these compounds remains confined to *in vitro* studies, lacking *in vivo* validation.

#### 3.4.2 Chloroquine

Since the late 1960s, the *in vitro* antiviral activity of chloroquine has been confirmed (145), demonstrating antiviral activity against a range of viruses such as Zika virus (146), dengue virus (147), and Ebola virus (148). Research indicates that the antimalarial drug amodiaquine selectively inhibits the replication of SFTSV, with a half-maximal effective concentration (EC_50_) of 19.1 ± 5.1 μM, which is comparable to that of favipiravir (EC_50_ =25.0 ± 9.3 μM) (149). Iodine-substituted derivatives (EC_50_ =15.6 ± 4.9 μM) effectively enhance its activity. However, amodiaquine’s insufficient antiviral activity hindered clinical development, necessitating structural modifications to identify potent derivatives. Researchers subsequently synthesized 98 novel derivatives of amodiaquine, among which compound C-90 demonstrated the highest *in vitro* activity (EC_50_ = 2.6 ± 0.6 μM). However, both C-90 and its hydrochloride salt demonstrated extremely low oral bioavailability in murine models. Subsequently developed hydrochloride derivatives C-A (EC_50_ =4.3 μM) and C-B (EC_50_ =8.2 μM) improved oral pharmacokinetic properties but exhibited weaker activity than C-90 (150). Certain derivatives of amodiaquine derivatives exhibit significant broad-spectrum antiviral efficacy. This series of compounds presents potential for clinical development as therapeutic agents against emerging viral infections and merits further investigation.

#### 3.4.3 Nitazoxanide

Following initial screening at maximum concentration (Cmax) doses and subsequent validation, researchers identified favipiravir, nitazoxanide, and peramivir as compounds exhibiting inhibitory effects on SFTSV replication (125). Among these, nitazoxanide—a broad-spectrum antiviral drug—has demonstrated efficacy against influenza viruses, SARS-CoV-2, Japanese encephalitis virus, and other pathogens. Notably, it potently inhibits SFTSV at concentrations below clinical plasma levels (Cmax =10 μg/mL), with an EC_50_ value of 0.57 μg/mL. These properties, combined with its convenient oral administration in influenza treatment, position it as a promising candidate for SFTSV therapy. However, the exact mechanism by which it acts against SFTSV remains to be clarified. Peramivir, an antiviral drug for influenza approved in South Korea, has an established clinical use basis. However, its mechanism of action against SFTSV may differ from that against influenza, necessitating further investigation (125).

#### 3.4.4 Hexachlorophene

After screening 1,528 FDA-approved drugs, researchers identified hexachlorophene as the most potent inhibitor of SFTSV replication (IC_50_ = 1.3–2.6 μM), demonstrating superior efficacy compared to known anti-SFTSV compounds such as ribavirin and favipiravir. Mechanistic investigations revealed that hexachlorophene does not interfere with viral attachment but inhibits entry by blocking membrane fusion. Molecular docking studies predicted its binding site as deep hydrophobic pocket (151) between domains I and III of the SFTSV Gc protein. This discovery facilitates the structural optimization of hexachlorophene to develop derivatives with enhanced activity and reduced toxicity. Furthermore, considering that hexachlorophene is an organochlorine compound extensively utilized for topical antimicrobial disinfection and agricultural sterilization (152), its disinfectant properties confer potential application value. It may be considered for environmental surface disinfection to reduce the risk of nosocomial outbreaks caused by SFTSV transmission via contaminated surfaces.

#### 3.4.5 Interferon

Interferon (IFN), as a widely expressed cytokine, possesses potent antiviral and immunomodulatory properties (153). Deficiency or suppression of type I IFN signaling increases susceptibility to SFTSV, resulting in elevated viral loads and exacerbated pathological damage (154). The activation of IFN signaling exhibits a dual nature (63, 155, 156). On one hand, its rapid and potent activation effectively suppresses early viral replication and promotes viral clearance, as demonstrated by the timely IFN/IRF pathway response observed in young ferrets (157). Conversely, excessive activation induces inflammatory storms and complement activation, exacerbating tissue damage and increasing lethality through the sustained upregulation of inflammatory immune responses. This is typically manifested by the abnormal production of chemokines that mediate leukocyte extravasation (46, 155, 158). This contradictory mechanism is particularly evident in aged ferret models, where delayed IFN/IRF responses hinder viral clearance, while persistent inflammation ultimately results in mortality (157). Specifically, during the late stages of SFTSV infection, IFN-α may drive uncontrolled inflammation, coagulation disorders, and multi-organ failure by excessively activating innate immunity and suppressing adaptive immunity (46). Conversely, high levels of IFN-γ within the thymus may induce thymic atrophy and impair T-cell development (159). Consequently, targeting interferon and its signaling pathways during the advanced stages of disease progression could potentially restore immune equilibrium. For instance, the JAK1/JAK2 inhibitor ruxolitinib has demonstrated preliminary evidence of enhancing survival rates and reducing the risk of ICU admission in critically ill patients (160).

Based on the key mechanism by which SFTSV achieves immune evasion through antagonism of the interferon response, interferon pathway modulators offer a therapeutic strategy targeting the host immune system. Bortezomib (PS-341) inhibits the NS-mediated degradation of RIG-I, thereby restoring upstream viral recognition mechanisms (161). Kaempferide enhances exogenous IFN efficacy and synergizes with virus-induced endogenous IFNs by amplifying downstream JAK/STAT signaling (162).

A deepening understanding of viral structure and pathogenic mechanisms further expands drug development approaches. For instance, targeting the interaction interface between viral NSs protein and host factors like STAT2 or STING (163), or intervening in key functional domains such as the PXXP motif (66) and conserved N-terminal sequence (164) of NSs, holds promise for blocking viral immune evasion. Conversely, enhancing the host’s innate immune regulatory capacity represents another critical strategy. Molecules such as SAFA (78) and DR1 (165) have been demonstrated to promote IFN expression, thereby elevating the overall immune response.

In summary, when administering targeted therapies against the interferon pathway and inflammatory cytokines, it is essential to carefully determine the optimal treatment timing while fully considering the dual effects of immune suppression and cytokine storms induced by SFTSV infection.

#### 3.4.6 Other immunomodulators

Current research on immunomodulators predominantly addresses the suppression of excessive immune activation and inflammatory damage. Glucocorticoids, widely utilized as immunosuppressive agents in clinical settings, exhibit controversial efficacy against SFTSV. While some studies affirm their effectiveness in mitigating excessive immune responses (169–171), a substantial body of research indicates that these drugs fail to significantly reduce patient mortality and may heighten the risk of adverse events, including secondary infections (166–168), thereby necessitating rigorous individualized risk-benefit assessments (172, 173). Additionally, intravenous immunoglobulin (IVIG) has not shown efficacy in reducing mortality or enhancing overall outcomes, warranting cautious clinical application (174).

In pursuit of more precise interventions, research has increasingly focused on immunomodulation targeting specific pathways. Interventions targeting cytokines have demonstrated potential. Early administration of anti-IL-6 antibodies after infection significantly improved survival rates in infected mice (175). Clinical studies suggest that the IL-6 receptor antagonist tocilizumab may help reduce patient mortality (176).

Modulation of inflammatory signaling pathways is also under investigation. Cyclophilin A (CyPA) has been found to promote the release of proinflammatory cytokines such as IL-6, IL-1β, and TNF-α. Its inhibitor, cyclosporine A, extended survival and reduced organ damage in IFNAR^-^/^-^ mouse models but failed to completely prevent death, indicating that its efficacy requires further optimization and validation (88). Additionally, viral NSs protein upregulates IL-10 by activating the host TPL2 signaling pathway, thereby suppressing immune responses. Inhibitors targeting this pathway have been shown to improve survival rates in infected mice (74).

### 3.5 Neutralizing antibody drugs

Neutralizing antibodies (nAbs) function as passive antiviral agents, contributing to prevention, therapeutic interventions, and the guidance of vaccine design (177). They impede viral entry by binding to functional entry molecules on the viral surface and elimination of infected cells through Fc-mediated *in vivo* effects (177). In convalescent serum from patients with SFTS, neutralizing antibodies can persist for up to a decade, although they exhibit considerable individual variability, thereby necessitating personalized monitoring (178).

Research indicates that the humoral immune response derived from convalescent patients primarily targets the highly immunogenic nucleocapsid protein. Despite the lack of direct neutralizing activity, these antibodies present potential utility for early diagnosis (179–181). The ¹¹¹In-labeled N protein monoclonal antibody probe ([¹¹¹In]In-DTPA-N-mAb) serves as a novel imaging probe. Through SPECT/CT, it facilitates non-invasive and precise visualization of SFTSV infection dynamics within the spleens of mice. This methodology holds significant promise as a powerful instrument for advancing the understanding of SFTSV pathogenesis, screening antiviral drugs, and achieving accurate clinical diagnosis and treatment (182).

The M segment of SFTSV encodes the glycoprotein precursor, which is cleaved into Gn and Gc (2). Gn plays a primary role in binding to host receptors (177). Structurally, the head of Gn adopts a compact triangular conformation, consisting of three subdomains (I, II, III) (183). Domain I (DI) serves as an ideal target for developing broad-spectrum neutralizing antibodies (184). Key neutralizing antibodies targeting DI include S2A5 (185), whose light chain complementarity-determining region L1 (CDR-L1) cross-linking mechanism enhances neutralizing efficacy. 40C10 (186) impedes viral internalization and demonstrates efficacy with delayed administration. Its humanized variant HAb-23 holds clinical potential (187). JK-8 achieves broad-spectrum protection at low doses by inhibiting Gn-CCR2 interaction (188).

Other neutralizing antibodies targeting Gn include human monoclonal antibodies hmAbs1F6, 1B2, and 4–5 derived from convalescent SFTS patient lymphocytes, which provide complete protection in murine models when used in combination (189). The bispecific antibodies bsAb1/bsAb3, which simultaneously target the Gn and Gc epitopes, exhibit neutralizing efficacy exceeding 100-fold higher than their parent antibodies and effectively inhibit viral escape modifications (184). The findings indicate that combination of antibodies targeting distinct epitopes might markedly enhance the antiviral activity of neutralizing antibodies. MAb 4-5, a humanized neutralizing monoclonal antibody derived from convalescent patients, specifically binds to the α6-helix of Domain III via its heavy chain CDR H3 to inhibit Gn-cell receptor interaction (183, 190). And Ab10 targets the DII and stem regions to inhibit membrane fusion. This antibody exhibits a strong affinity for its targets and is efficacious against the vast majority of epidemic strains, with *in vitro* neutralizing activity superior to that of antibody MAb4-5 (191).

In addition to the aforementioned antibodies, the production of highly effective antiviral antibodies utilizing various advanced technology platforms has shown considerable therapeutic promise and intriguing applications. The mRNA-LNP-delivered S/A-TEN antibody can be swiftly synthesized, and a low-dose, two-dose strategy provides 100% protection across multiple models while widely neutralizing viruses of different genotypes (192). Through high-throughput nanobody screening utilizing next-generation sequencing (NGS) and proteomics on camels immunized with Gn protein, a total of 19 candidate nanobody sequences were identified. Six of these were chosen for recombinant expression and purification, among which nanobody 57493 demonstrated strong affinity and specificity toward Gn protein (193). Another camel-derived single-domain antibody, SNB02, demonstrates exceptional efficacy both *in vitro* and *in vivo*. It not only suppresses viral replication but also alleviates infection-induced thrombocytopenia and multi-organ damage, demonstrating interesting application prospects (194).

In summary, neutralizing antibodies demonstrate multifaceted application potential: in SFTSV diagnosis, they concentrate on nucleocapsid protein applications; in therapeutics, they facilitate broad-spectrum neutralization by targeting glycoproteins (**Table 1**) and in pathological research, they elucidate infection mechanisms through *in vivo* dynamic tracing. Nonetheless, none of these therapeutic antibodies have undergone clinical confirmation, and their actual efficacy and safety necessitate comprehensive assessment.

**Table 1:** Results of *in vitro* and *in vivo* neutralizing antibody assays for SFTSV.

Antibody	Source of antibodies	Target site	Affinity	Primarily involves viral strains	Neutralization test	Animal model	Animal experimental strains and infection levels	Intravenous dosing	Prevention/post-exposure administration survival rate	Advantages/Limitations	Reference
MAb 4-5	Lymphocytes from 5 SFTS Recovery Patients	A linear epitope in the extracellular domain (aa 20–452) of the Gn glycoprotein	\	JS-2010-003	The concentration required for 50% neutralization of 100 TCID50 SFTSV is approximately 2.0 μg/ml.	\	\	\	\	Neutralizes a wide range of strains isolated from multiple regions in China at a concentration of 5 μg/mL.	2013/7/10 (190)
	Peripheral blood mononuclear cells from a recovered SFTS patient	The α6 helix of domain III of the Gn	KD=25.9 nM	SDYY007	The concentration required to neutralize 100 TCID50 of SFTSV is 44.2 μg/mL	\	\	\	\	No binding or neutralizing activity against RVFV	2017/7/25 (183)
Ab10	Peripheral blood mononuclear cells from a recovered SFTS patient	Gn Domain II,Stem region	KD=104 pM Kon=7.4 × 10^5^ M^-^¹s^-^¹ Koff=7.7 × 10^-5^ s^-^¹	Gangwon/Korea/2012	50 µg/mL reduced the proportion of infected cells from 100% to 5.6%.	A129 mice (IFNAR1-deficient)	Gangwon/Korea/2012 (Subcutaneous injection of 2 PFU or 20 PFU)	Intraperitoneal injection of 600 µg/day (approximately 30 mg/kg) for 4 consecutive days	+1h: 100% survival +3 days: 100% survival (2 PFU), 80% survival (20 PFU) +5 days: 80% survival (2 PFU)	For conservative non-linear epitopes, *in vitro* neutralizing activity significantly outperforms that of the known antibody MAb4-5.	2019/2/1 (191)
mRNA S/A-TEN	Ab10 antibody sequence, expressed via mRNA-LNP technology	\	\	JJ strain HB29 strain GW strain	The highest FRNT50 titer was observed at a 5 mpk dose.	IFNAR Ab Mice/IFNAR-1 KO Mice	JJ strain (IFNAR Ab Mice 10^5^FFU/ 10^6^FFU, IFNAR-1 KO Mice: 10³ FFU)	Beginning 14 hours after infection, Intravenous injection, every 3 days, total of 2 doses Dosage: 3 mpk (60 µg)	IFNAR-1 KO Mice 100% Survival Rate (5/5)	Highly efficient expression, convenient production, with dosage optimization requiring refinement.	2025/4/1 (192)
SNB02	Immune Camel	Gn	*KD* = 5.479×10^-9^M	E-JS-2013-24	Immunofluorescence (Vero E6) or qRT-PCR (PBMCs) IC_50_:=1.05 μg/mL in Vero E6, < 2.7μg/mL in PBMCs	NCG-HuPBL Humanized Mice	E-JS-2013-24 (2 × 10^5^ TCID_50_)	400 μg per mouse, intraperitoneal injection	+1h: Viral load reduction > 2 log_10_ +24h: Viral load reduction 1 log_10_	High thermal stability, low immunogenicity but short half-life, narrow therapeutic window	2020/7/9 (194)
Nanobody 57493	Immune Camel	Gn	\	\	ELISA: 3.7 nM binding	\	\	\	\	High-throughput screening, but insufficient research data	2022/9/24 (193)
SF5	Memory B cells isolated from peripheral blood mononuclear cells of a recovered SFTS patient	Gn Domain I	KD= 339.3± 44.32 nM	HB29 VSV-based pseudovirus	IC_50_= 149.1 ± 30.3 μg/mL (Pseudovirus) 73.2 ± 13.8μg/mL (True Virus)	IFNAR1-/-A129 mice (100 TCID_50_)	HB29 (100 TCID_50_)	Subcutaneous injection doses:2.5, 5, 10 mg/kg	-24h:5mg/kg:100% survival +24h:2.5mg/kg:25% survival 5mg/kg:80% survival 10mg/kg:100% survival +48h/+72h: 10mg/kg:100% survival	Strong *in vivo* protective efficacy but low affinity, leading to escape mutations	2024/6/3 (184)
SF83		Gc Domain II	KD = 4.78 ± 2.50 nM		IC_50_= 3.49 ± 0.38 μg/mL (pseudovirus) 29.5 ± 12.8 μg/ml (True virus)			Subcutaneous injection doses: 10,20mg/kg	-24h: 10mg/kg:0% survival 20mg/kg:40% survival +24h:10mg/kg:0%	Strong *in vitro* neutralizing activity, but weak *in vivo* protective efficacy	
bsAb1/bsAb3	Based on SF5 and SF83	Gn domain I Gc domain II	Binding to Gn: bsAb1:215 ± 15.9nM bsAb3:258 ± 24.4 nM Binding to Gc: bsAb1:3.38 ± 0.09 nM bsAb3:4.54 ± 0.53 nM		IC_50_= 0.16–0.78 µg/mL (Pseudovirus) IC_50_= 1.05–1.72 µg/mL (True virus)			Subcutaneous injection doses:2.5, 5, 10 mg/kg	-24h:5mg/kg:100% survival +24h:2.5 mg/kg:40% 5 mg/kg:100% 10 mg/kg:100% +48h/+72h:10mg/kg:100%	Synergistic neutralization, reduction of escape mutations, and sequence assembly will affect activity	
40C10	immunized BALB/c mice	Gn Spatial Conformation Position	\	HBZN15 HBGS13 HBMC5 WCH	IC_50_= C1: 2.015 ng/mL C2: 7.506 ng/mL C3: 5.940 ng/mL C4: 4.360 ng/mL	IFNAR^-^/^-^ C57BL/6 mice	HBGS13 (C2) HBMC5 (C3) WCH (C4) dose: 20LD_50_/50LD_50_	Intraperitoneal injection Dose: 30μg/g	20 LD_50_: +1–4 days: 100% survival 50 LD_50_: +1–2 days: 100% survival; +3 days: 83.3%; +4 days: 33.3%	Neutralizes SFTSV of different genotypes and GTV, HRTV, but requires multiple injections and is not humanized.	2024/6/7 (186)
	immunized BALB/c mice	Gn Domain I	KD = 1.35 × 10^-10^ M	C3 genotype (GenBank: QNR55516.1, AQX34644.1)	IC_50_=9.19 ng/mL	\	\	\	\	New tab position but minimum effective dose not determined	2024/9/1 (187)
	Humanized Antibody (HAb-23)		KD = 2.03 × 10^-10^ M		IC_50_= 17.36 ng/mL	IFNAR^-^/^-^ C57BL/6 mice	HBGS13 (C2) dose: 50 LD_50_	Intraperitoneal injection Dose: 30μg/g (approximately equivalent to30mg/kg)	-24h:100% survival +24h:100% survival		
S2A5	Immunized BALB/c mice	Gn Domain I	KD = 3.25 nM	WCH97 QD02	pseudovirus: IC_50_< 0.6239 μg/mL QD02: 0.0402 μg/mL WCH97: 0.0228 μg/mL	IFN-α/βR^-^/^-^ mice	HBMCS (D) dose: 500 TCID_50_	Single intraperitoneal injection Dose: 400 μg	-24h:100% survival +6 hours: 100% survival +24hours:100% survival +48 hours: 33% survival	Broad-spectrum neutralizing activity (covering genotypes A–F), providing complete protection with a single dose while inhibiting viral attachment and membrane fusion	2024/8/15 (185)
JK-8	Memory B cells isolated from peripheral blood mononuclear cells of four SFTS survivors	Gn Domain I	KD = 1.0 pM	HBMCl6	FRNT_50_ <100 ng/mL	IFNAR1^-^/^-^ C57BL/6 mice	HBMCl6 (Clade I) dose: 20 FFU	Single intraperitoneal injection Dose: 10 μg	-24h:100% survival +1/2days: 100% survival +3 days: 62.5% survival	Broad-spectrum neutralizing activity (covering 5 evolutionary branches), providing complete protection with a single low dose, Sample sources limited to 4 recovered patients, not covering all viral evolutionary branches	2025/1/1 (188)

## 4 Future research directions

Although drugs such as ribavirin, favipiravir, calcium channel blockers, nucleoside analogues, and interferons have demonstrated potential in treating SFTSV infection (**Tables 2** and **3**), their clinical efficacy is constrained by multiple factors. These factors encompass critical timing of administration, viral load levels, the severity of the patient’s condition, and the side effects of the medications themselves. These limitations hinder current therapies from effectively addressing the significant treatment challenges presented by the elevated mortality rate of SFTS and its intricate pathophysiological underpinnings.

**Table 2:** *In vitro* activity data of candidate drugs against SFTSV.

Drug name	Virus strain	Cell line	CC_50_	Assay method	IC_50_/EC_50_/EC_90_	SI	Reference
Ribavirin	HB29	Vero	>320 μM	Virus Yield Reduction Assay	EC_90_:49.7± 4.0μM	>6	(135)
	HB29	Vero	\	Indirect Immunofluorescence Assay	EC_99 :_64 ± 17μg/ml(263 ± 68μM)	\	(96)
		Huh7	\		EC_99 :_20 ± 5μg/ml(82 ± 20μM)	\	
		U2OS	\		EC_99 :_19 ± 2μg/ml(78 ± 6μM)	\	
	SPL030	Vero	\		EC_99_:104 ± 22μg/ml(424 ± 88μM)	\	
		Huh7	\		EC_99 :_15 ± 2μg/ml(63 ± 7μM)	\	
		U2OS	\		EC_99 :_19 ± 4μg/ml(73 ± 15μM)	\	
T-705	YG1	Huh-7	\	Immunofocus Assay	IC_50_:5.62μM	\	(134)
	clinical isolate	Vero	>50μM	RT-PCR	EC_50_:4.1 ± 0.6μM	\	(150)
	KADGH/2013/Korea	Vero E6	\	RT-PCR	EC_50_: 6.7μM	\	(125)
				focus forming assay	EC_50_: 4.14μM	\	
	Laboratory-isolated strains (GenBank: MZ561690.1, MZ561691.1,MZ561692.1)	Huh7	>100μM	CPE-based cell viability Assay	EC_50_:11.41 ± 7.83μM	>8.76	(221)
Benidipine Hydrochloride	HBMC16_human_2015	Vero	96.92μM	qRT-PCR	IC_50_:1.412 μM	68.6	(131)
Nifedipine	\		>250μM		IC_50_:98 μM	\	
Loperamide	YG1	Huh-7	\	Immunofocus Assay	IC_50_:4.4 μM	\	(133)
Manidipine	YG1	SW13	57.03μM	Cell viability assay	IC_50_:2.83 μM	20.15	(134)
		Huh-7	28.2μM		IC_50_:3.17 μM	8.19	
2’-Fluoro-2’-deoxycytidine	HB29	Vero	> 320 μM	Virus Yield Reduction Assay	EC_90_:3.7 ± 2.0μM	>86	(135)
4-FIU	HBMC16_human_2015	Vero	>1000 μM	Luciferase Activity (SFTSV-Nluc)	IC_50_:5.499μM	\	(136)
VV261	HBMC16	Vero	> 100 μM	RT-qPCR	EC_50_:0.89 ± 0.23 μM	> 112	(137)
Fludarabine	SFTSV-A(JS2010-14)	Vero	3.10 ± 0.20μM	qRT-PCR	IC_50_:0.83 ± 0.03 µM	\	(139)
		BHK21	3.61 ± 0.07μM		IC_50_:0.27 ± 0.001 µM	\	
		MG	6.21 ± 1.30μM		IC_50_:0.28 ± 0.17 µM	\	
		HMC3	12.68 ± 2.30μM		IC_50_:0.42 ± 0.01µM	\	
	SFTSV-E(JS2014-16)	Vero	3.10 ± 0.20μM		IC_50_:0.31 ± 0.02µM	\	
Caffeic acid	YG1	Huh7.5.1–8	7.6 mM	qRT-PCR	Virus co-cultured with CA IC_50_:0.048 mM	158	(140)
					Virus pre-incubated with CA for 4 hours IC_50_:0.019 mM	400	
					Drug added post-viral infection (MOI = 0.01) IC_50_:0.18 mM	42	
					Drug added post-viral infection (MOI = 1) IC_50_:>1 mM	<7.6	
Amodiaquine	clinical isolate	Vero	>50μM	qRT-PCR	EC_50_:19.1 ± 5.1μM	\	(150)
Nitazoxanide	KADGH/2013/Korea	Vero E6	\	qRT-PCR	EC_50_: 2.3 µg/mL	\	(125)
			\	Plaque Assay	EC_50_:0.57 µg/mL	\	
Peramivir			\	RT-PCR	EC_50_: 25.4 µg/mL	\	
			\	Plaque Assay	EC_50_: 12.9 µg/mL	\	
Hexachlorophene	HB29	Vero	24.3 ± 3.2μM	RT-PCR	IC_50_: 1.3 ± 0.3	18.7	(151)
				Plaque Assay	IC_50_:2.6 ± 0.14 μM	\	
Baloxavir acid	Rescuing in 293T cells via a reverse genetics system	Vero E6	\	FRET-based endonuclease Assay	IC_50_:135 ± 5 nM	\	(201)
				Plaque reduction Assay	EC_50_:263 ± 14 nM		
Tanshinone I	HBMC16	Vero	245μM	qRT-PCR	EC_50_:2.03μM	121	(195)
		Huh-7	\		EC_50_:0.44μM	\	
Tanshinone IIA		Vero	241.7μM	qRT-PCR	EC_50_: 2.86μM	85	
Licoflavone C	HBMC16	Vero	>300μM	qRT-PCR	EC_50_:1.85 μM	>162.16	(203)
	SFTSV CEN	\	\	FRET (Enzymatic Activity)	IC_50_:35.5μM	\	
SGc1	JS-2010-014	L02	>400 µM	qRT-PCR	IC_50_: 2.45 ± 0.53 μM	\	(213)
		Vero			IC_50_: 5.61 ± 0.51 μM	\	
		BHK21			IC_50_: 4.12 ± 0.09 μM	\	
SGc8		L02	>400 μM		IC_50_: 3.44 ± 0.47 μM	\	
		Vero			IC_50_: 4.04 ± 0.53 μM	\	
		BHK21			IC_50_: 8.86 ± 1.02 μM	\	
HKU-P1	HB29	Huh-7	>1000μg/mL	RT-qPCR	IC_50_:523.9 μg/mL	\	(211)
WYFA15	HBMC16	Huh-7	181.3μM (134.9–318.4μM)	RT-qPCR(Immunological focus assay)	IC_50_:7.28 (6.16–8.58) μM	24.9	(212)
		HepG2	\		IC_50_:18.8μM	\	
CP-COV03	NCCP43270	Vero	\	RT-qPCR	IC_50_ <0.125 µM	\	(198)
				Plaque reduction Assay	IC_50_:1.893 µM		
Bazedoxifene acetate	Laboratory-isolated strains, (GenBank: MZ561690.1, MZ561691.1, MZ561692.1)	Huh7	3.81 ± 1.67μM	CPE-based cell viability Assay	EC_50_:0.35 ± 0.09µM	10.89	(221)
Tilorone	\	Huh7	\	CPE-based cell viability Assay	EC_50_:0.42 ± 0.02μmol/L	≈23.81	(235)

**Table 3:** Efficacy evaluation of anti-SFTSV drugs in infected animal models.

Drug name	Drug type	Targeted	Mechanism of action	Animal models	Virus strain	Route of exposure/Dosage	Experimental design	Mortality/Survival rate	Reference
Ribavirin	Nucleoside analogues	Virus targeting	Inhibit viral RNA polymerase	IFNAR1-/-A129 mice	YG-1	Subcutaneous (10^6^ ffu)	Intraperitoneal injection at 1-, 24-, 48-, and 72-hours post-infection Dosage: 2 mg per animal	Mortality Rate:80% (4/5)	(100)
				STAT2 KO Hamster	HB29	Subcutaneous (50 PFU)	Begin oral administration 1 day after infection, twice daily for a total of 10 days. Dosage: 75 mg/kg/day	Mortality Rate:100% (5/5)	(120)
Favipiravir	Nucleoside analogues	Virus targeting	Inhibit viral RNA polymerase	IFNAR−/− C57BL/6 mice	SPL010	Subcutaneous (10^6^ TCID_50_)	Oral administration begins 1 hour to 5 days after infection and continues for 5 consecutive days. Dosage: 120 or 200 mg/kg/day, administered in two divided doses.	120 mg/kg/day group 1–3 days post-infection: 100% survival 4 days: 67% survival 200 mg/kg/day group 1–4 days post-infection: 100% survival 5 days: Partial mortality	(119)
				STAT2 KO Hamster	HB29	Subcutaneous (50 PFU)	Start oral administration one day after infection, twice a day, for a total of 10 days Dosage: 300 or 150 mg/kg per day	100% survival	(120)
Benidipine hydrochloride	Calcium Channel Blockers	Host targeting	Lowering intracellular Ca^2+^ levels inhibit viral replication.	Humanized mouse model	HBMC16_human_2015	Intraperitoneal (10^5^ TCID_50_)	Administer via gastric lavage 1 hour after poisoning, twice daily for 7 days. Dosage: 15 mg/kg/day	Mortality Rate 16.7% (1/6)	(131)
Nifedipine							Administer via gastric lavage 1 hour after poisoning, twice daily for 7 days. Dosage: 100 mg/kg/day	Mortality Rate 0% (0/6)	
manidipine			Inhibits calcium ion influx and suppresses calcineurin activation. Inhibits G-actin formation. Blocks SFTSV nucleoprotein-induced inclusion body formation.	IFNAR−/− C57BL/6 mice	YG-1	Subcutaneous (10 FFU)	Intraperitoneal injection on days 4 and 5 post-infection, twice daily Dosage: 10 mg/kg	Mortality rate reduction	(134)
2’-Fluoro-2’-deoxycytidine	Nucleoside analogues	Virus targeting	\	IFNAR^-^/^-^ mice	HB29	Subcutaneous (3 PFU)	Intraperitoneal injection, twice daily for 8 days Dosage: 50, 100, 200 mg/kg/day	50mg/kg/day:80% survival 100mg/kg/day:100% protective 200mg/kg/day:80% survival	(135)
4-FIU	Nucleoside analogues	Virus targeting	\	IFNAR1-/-A129 mice	HBMC16_human_2015 SFTSV-Nluc	Intraperitoneal (1000 PFU SFTSVwt/10,000 PFU SFTSV-Nluc)	Administer via gastric lavage once daily after infection Dosage: 20 mg/kg	100% survival	(136)
VV261	Nucleoside analog bis-prodrug	Virus targeting	\	IFNAR1-/-A129 mice	HBMC16	Intraperitoneal (1000 PFU)	Oral treatment begins 1 hour after poisoning. Administer once daily for 7 consecutive days. Dosage: 2.5, 5, or 10 mg/kg	2.5 mg/kg:16.7% survival 5 mg/kg:100% survival 10 mg/kg:100% survival	(137)
Cyclosporine A	Immunomodulator	Host targeting	Inhibits extracellular CyPA (eCyPA) from binding to the host cell surface receptor CD147, thereby blocking the activation of the MAPK signaling pathway. Reduces the release of pro-inflammatory cytokines.	IFNAR−/− C57BL/6 mice	JS14	Subcutaneous (10^6^ TCID_50_)	Administration begins 1 hour after infection and continues for 5 consecutive days. Intraperitoneal injection Dosage: 10 mg/kg/day	The time to death was delayed to days 7, 8, and 9 post-infections (with the final case fatality rate remaining at 100%)	(88)
Tanshinone I/IIA	Natural compounds	Virus targeting	Inhibition of the enzymatic activity of cap-dependent endonuclease (EndoN)	C57BL/6J mice	HBMC16	Intraperitoneal (5×10^5^ FFUs)	Tanshinone I: Intravenous:20mg/kg intraperitoneal:20 mg/kg intragastric:20 mg/kg Tanshinone IIA:intravenous:50 mg/kg	No significant toxicity was observed	(195)
Licoflavone C	Natural flavonoid compounds	Virus targeting	By disrupting the active conformation of SFTSV CEN, non-competitive inhibition of the substrate is induced.	C57BL/6J mice	HBMC16	Intraperitoneal (10^4^ FFU/10^5^ FFU)	Intravenous injection 2 hours after poisoning, every 12 hours for two days Dosage: 5/10/20 mg/kg	\	(203)
WYFA15	Small-molecule inhibitor	Host targeting	Inhibition of SMS1 activity reduces the synthesis of SM(d18:1/16:1)	C57BL/6J mice (pretreated with anti-IFNAR1 antibody)	HBMC16	Intraperitoneal (2×10³ FFU)	Intraperitoneal injection, initiated 1 hour after infection, administered for 5 days Dosage: 100 mg/kg/bid	Mortality Rate 26.7% (4/15)	(212)
Anidulafungin	Antifungal drugs	Host targeting	Interfering with clathrin-mediated endocytosis inhibits viral entry into cells	IFNAR1-/-A129 mice	WCH-2011/HN/China/isolate97	Intraperitoneal (1 LD_50_/10 LD_50_)	Intraperitoneal injections began 1 hour after challenge and continued for 14 days Delayed treatment group: Administered on days 1, 2, and 3 post-infection Dose: 10 mg/kg/day	1 LD_50_: 100% survival 10 LD_50_: 66.7% survival Delayed administration(1 LD_50_): Survival rate 100% when administered within 48 hours post-infection; drops to 50% when administered at 72 hours.	(219)
Toosendanin	Natural compounds	Host targeting	\	C57BL/6 mice anti-IFNAR1-treated C57BL/6 mice	HBMC16	Intraperitoneal injection: C57BL/6 mice (10^5^ FFU) Anti-IFNAR1-treated C57BL/6 mice (2000 FFU)	C57BL/6 mice: Intraperitoneal injections initiated 3 days prior to infection and continued for 3 days post-infection. Dose: 1 mg/kg/day Anti-IFNAR1-treated C57BL/6 mice: Intraperitoneal injections initiated 1 hour post-infection. Dose: 4 mg/kg/day	\	(220)
Bazedoxifene acetate (BZA)	Estrogen Receptor Modulators	Host targeting	Activate the host’s innate immune response and regulate the expression of genes such as GRASLND, CYP1A1, TMEM45B, and TXNIP	ICR suckling mice	SFTSV (GenBankMZ561690.1, MZ561691.1, MZ561692.1)	Intraperitoneal injection (1.25×10^6^ PFU)	Beginning 4 hours post-infection, administer intraperitoneal injections (20, 10, 5 mg/kg) daily for 7 consecutive days; Oral administration to female mice (20 mg/kg)	The survival rate in the 10 mg/kg group was 90.9%. The survival rate in the 20 mg/kg group (administered via the mother mouse) was 72.7%.	(221)
Tilorone	Small-molecule immunomodulators	Host targeting	Activate the host innate immune response (via the RIG-I pathway) and promote the production of type I interferons (IFN-α/β).	ICR suckling mice, IFNAR1-/-A129 mice BALB/c mice	\	ICR mice: Intracerebral injection of 5×10³ PFU A129 mice: Intraperitoneal injection of 10 PFU	Treatment group: Intraperitoneal injection 1–7 days post-infection Dose: 20 mg/kg/day Prevention group: Intraperitoneal injection for 3 consecutive days prior to infection Dose: 25/50 mg/kg/day	Treatment group with ICR mice: Survival rate 78.94% Prevention group: 50 mg/kg survival rate 70% 25 mg/kg survival rate 53.33% A129 mice: Mortality rate 100%	(235)
Metformin	Oral hypoglycemic agents (biguanides)	Host targeting	Activate AMPK, inhibit mTOR, suppress autophagy	BKS-db/db diabetic mice (female) (pre-treated with anti-IFNAR1 antibody)	HBMC16_human_2015	Intraperitoneal injection (4 × 10^4^ PFU per mouse)	5 days prior to infection to 5 days after infection, Dose: 300 mg/kg/day (administered via gastric tube)	Mortality Rate: 40% (4/10)	(228)

In recent years, the application of new technologies has significantly accelerated the investigation of antiviral drugs aimed at SFTSV, substantially expanding the pathways for drug screening and development. Meanwhile, comprehensive research into the pathogenic mechanisms of SFTSV and pathological alterations in patients are continually revealing new, more promising therapeutic targets and intervention options. This has propelled the development of broad-spectrum antiviral drugs targeting conserved functional areas and universal mechanisms of viruses (195–197), while also facilitating the creation of highly effective and specific therapeutic medications.

**Figure 1:**
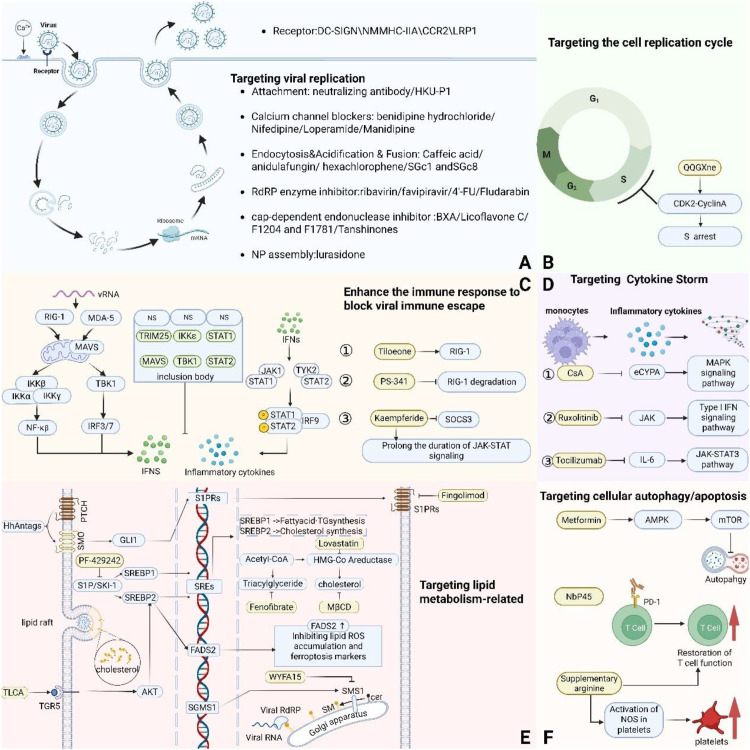
Schematic diagram of the mechanism of action of SFTSV antiviral drugs. **(A)** Viral replication involves stages including attachment, endocytosis, replication, assembly, and release. Neutralizing antibodies and HKU-P1 block viral adsorption; calcium channel blockers inhibit invasion and replication by regulating Ca^2+^ influx; compounds such as caffeic acid and anifentanyl interfere with endocytosis and membrane fusion; ribavirin and favipiravir inhibit RdRp activity; baloxavir marboxil and luteolin C block cap-snatching; and lurasidone binds NP to disrupt RNP function. **(B)** QQGX targets the CDK2-CCNA2 complex via luteolin, inducing S-phase arrest and inhibiting viral replication. **(C)** RIG-I/MDA-5 recognizes vRNA, activates NF-κB and IRF3/7 pathways via MAVS, inducing IFN-α/β production. IFN activates the JAK-STAT pathway through IFNAR, expressing ISGs to establish an antiviral state. SFTSV NSs forms inclusion bodies that inhibit these pathways, evading immunity. Tilorone activates RIG-I to enhance IFN-I production; PS-341 inhibits proteasome activity to prevent NSs degradation of RIG-I; kaempferol prolongs JAK-STAT signaling to boost ISG expression. **(D)** Cytokine storms arise from the excessive release of inflammatory factors by overactivated immune cells. CsA inhibits eCypA to block the MAPK pathway; Ruxolitinib suppresses the JAK pathway; Tocilizumab blocks the IL-6–JAK-STAT3 pathway, thereby alleviating the storm. **(E)** Targeting lipid metabolism: HhAntag inhibits viral protein synthesis via the GLI1-S1PR axis and Fingolimod directly acts on S1PR; WYFA15 inhibits SMS1; Lovastatin and Fenofibrate selectively suppress cholesterol and triglyceride synthesis, respectively; TLCA activates the TGR5-PI3K/AKT-SREBP2 axis, upregulates FADS2 to inhibit lipid ROS and ferroptosis. **(F)** Targeting autophagy/apoptosis: Metformin inhibits autophagy via mTOR; NbP45 blocks PD-1 to restore T cell function; Arginine enhances platelet and T cell function via NOS. The figure was created with BioRender.com.

Further investigation into the antiviral mechanisms of action and critical structural features of existing and candidate drugs not only aids in understanding drug efficacy, guiding the optimization of existing drugs and the design of novel lead compounds (133, 142, 197), but also establishes the theoretical basis for the systematic design of combination therapy protocols. **Figure 1** summarizes the major drugs discussed in this paper and provides schematic diagrams of their target sites. Additionally, the application of innovative nanodelivery technologies shows potential for improving drug targeting, stability, and bioavailability, thereby overcoming delivery challenges (198, 199).

In the future, the integration of precision targeting and combination therapy strategies targeting key viral enzymes, host factors, and immune regulation, combined with the advancement of novel delivery technologies and vaccine platforms, will create new avenues to overcome existing challenges in viral therapeutics.

### 4.1 Screening of targeted inhibitors based on SFTSV key enzymes

The N-terminal endonuclease (endoN) of the L protein in SFTSV cleaves host mRNA through a “cap-snatching” mechanism to obtain the cap structure and initiate viral transcription (200). This endonuclease relies on Mn^2+^ as a cofactor, and mutations in critical catalytic residues (e.g., H80A, D112A, etc.) significantly diminish its enzymatic activity. Its α/β mixed-fold structure resembles that of most **s**egmented Negative-sense RNA virus (sNSV) endonucleases, with the unique features including an N-terminal Beta-Cap responsible for maintaining structural stability and a C-terminal Dynamic α6 Helix associated with enzyme activity regulation (201). Due to endonuclease N’s critical function in viral replication and its conservation in sNSVs, it has emerged as a significant antiviral target. The influenza drug baloxavir marboxil (BXA) (201) and benzothiazole compounds (such as F1204 and F1781) (202) efficiently suppress SFTSV endonuclease activity by targeting metal ions at the endonuclease active site. The novel inhibitors Tanshinone I/IIA impede cap-cleavage by associating with the endonuclease domain (195). Licorice flavonoid C disrupts the active conformation through hydrogen bonding and hydrophobic interactions, noncompetitively inhibiting RNA cleavage (203). Both compounds demonstrate anti-SFTSV activity *in vitro* and *in vivo*.

Additionally, the NP encoded by the SFTSV S segment is involved in the release, replication, and assembly processes of the viral RNP complex. Due to its similarly conserved function, the N protein may likewise represent a potential target (204). lurasidone interferes with genomic replication by binding to NP (205). The host factor Moloney leukemia virus 10 protein (MOV10) directly inhibits RNP assembly by targeting the N-arm domain of the NP. This action is notably independent of MOV10’s RNA helicase function and the interferon pathway (206). Another host factor, the Myxovirus resistance protein A (MxA), inhibits RNP activity by disrupting the interaction between the viral NP and the RdRp (207). The mechanisms of action of the aforementioned host factors provide potential new targets for pharmacological research.

### 4.2 Antiviral peptides and small molecule drug design based on structural biology

Antiviral peptides are naturally occurring or synthetically engineered small-molecule peptides that exert broad-spectrum antiviral effects by disrupting viral particles, obstructing viral entry, and inhibiting replication. They represent significant candidates for innovative antiviral pharmaceuticals. Structure-guided research demonstrates significant application potential. The structure-based virtual screening and validation process generally utilizes methodologies such as molecular dynamics simulations, alanine scanning, and peptide docking to identify potential inhibitors of SFTSV binding sites from FDA-approved drug libraries or established compound databases, or to design and synthesize novel antiviral peptides/small molecule compounds (205, 208–210). Subsequently, the intricate architectures of candidate compounds targeting viruses are clarified by structural biology methodologies, including X-ray crystallography and cryo-electron microscopy, which offer validation of interactions and atomic-level specifics. These high-resolution structural insights allow for in-depth analysis and identification of critical binding residues, which can inform therapeutic optimization to enhance potency (211, 212), thereby significantly decreasing the time and financial expenditures associated with drug development.

The computer-aided designed cyclic peptide HKU-P1 effectively binds to the SFTSV Gn protein, exerting a neutralizing effect during the viral entry phase. Its combination with favipiravir demonstrates synergistic antiviral activity. However, it exhibits typical constraints of peptide molecules, including low oral bioavailability, short half-life, and suboptimal ADME (Absorption, Distribution, Metabolism, Elimination) properties (211). Alongside methods aimed against the Gn protein, the antiviral peptides SGc1 and SGc8, derived from the viral Gc protein impede viral entrance by obstructing membrane fusion. They exhibit potent antiviral activity (IC_50_ < 10 μM) in L02, Vero, and BHK21 cells, alongside minimal cytotoxicity, low immunogenicity, and robust stability within the temperature range of -20 °C and 37 °C. To alleviate protease degradation risks, SGc1 and SGc8 underwent N-acetylation and C-amidation modifications. However, although these modifications provided some advantages, they did not address the problem of low permeability. Furthermore, critical parameters such as the *in vivo* distribution and actual half-life of the modified peptides have yet to be evaluated in *in vivo* systems, like animal models (213). Research on the viral replication phase reveals that sphingomyelin synthase SMS1 catalyzes the synthesis of sphingomyelin SM (d18:1/16:1) within the Golgi apparatus. This sphingomyelin directly interacts with the helical-turn-helical motif of the viral RdRp protein, facilitating the formation of the viral replication complex. Studies indicate that increased SMS1 expression is associated with a heightened risk of serious disease in elderly individuals. WYFA15, an inhibitor designed to target SMS1 and optimized via molecular docking, effectively impeded the replication of several RNA viruses in both cellular and animal models while demonstrating favorable safety profile (212).

The profound integration of artificial intelligence (AI) with structural biology technologies holds promise for further advancing the optimization and development of peptide molecules and small-molecule inhibitors. It serves a crucial function in essential phases such as target identification, virtual screening, *de novo* design, ADMET (absorption, distribution, metabolism, excretion, and toxicity) and to prediction, synthetic planning, and automated synthesis, significantly accelerating the drug development process (214).

### 4.3 Precise screening of key host factors using the CRISPR/Cas system

Clustered Regularly Interspaced Short Palindromic Repeats (CRISPR) technology has been instrumental in identifying systemic host factors, confirming targets, clarifying mechanisms, and revolutionizing diagnostic methodologies. Through genome-wide CRISPR knockout screening, researchers have identified essential host factors, including LRP1 (54), SMS1 (212), and CCR2 (53). Antagonists targeting these host factors have demonstrated antiviral effects. CRISPR-engineered gene knockout cell models not only validate target functions but also provide foundational tools for in- comprehensive research of infection mechanisms. The A549 cell line, established by Dr. Liu’s team through CRISPR/Cas9 technology to knock out the clathrin heavy chain (CLTC), confirms that lipid raft-mediated endocytosis may be a key entry pathway for SFTSV (215). Furthermore, CRISPR-based validation revealed the essential role of p38α in viral replication, with its inhibitor SB203580 capable of reducing SFTSV viral RNA levels and infectious viral particle production (87). CRISPR technology has spawned multiple diagnostic applications. A dual-gene, single-tube detection system based on Cas12a/Cas13a enables highly sensitive and rapid on-site SFTSV diagnosis (216). The CasFAS live-cell RNA imaging system facilitates dynamic monitoring of viral RNA (217).

### 4.4 Antiviral strategy based on synergistic action targeting both virus and host

Antiviral medications can be roughly classified into two primary categories depending on their mechanisms: Virus-targeting antivirals (VTAs) and host-targeting antivirals (HTAs) (218). VTAs directly or indirectly target viral components, exerting effects by inhibiting viral entry, replication, or assembly, such as the key enzyme inhibitors and antiviral peptides previously outlined. HTAs target host proteins essential for the viral lifecycle, indirectly inhibiting viral proliferation by regulating host cellular processes (e.g., signaling pathways) or immune system functions. Anidulafungin obstructs viral internalization during the viral invasion phase by inhibiting the clathrin-mediated endocytosis pathway (219). Toosendanin targets the viral internalization process to exert its impact. The lack of resistance development after serial passage suggests it may achieve potent inhibition by regulating host factors such as calcium channels (220). Upon virus entry into the replication phase, badoxifene acetate (BZA) inhibits viral replication by upregulating the expression of the host genes GRASLND and CYP1A1 (221). Meanwhile, the Hedgehog pathway inhibitor HhAntag suppresses viral protein translation by downregulating GLI1 protein, which in turn downregulates the expression of its downstream targets S1PR1 and S1PR5 (222). Furthermore, the active component luteolin in the traditional Chinese medicine compound Qingqi Gushe Decoction (QQGX) effectively inhibits SFTSV replication by inducing cell S-phase arrest through targeting the CDK2-CCNA2 complex (223). Notably, the mechanism of action of the nanobody NbP45 centers on immune modulation. By blocking the PD-1/PD-L1 immune checkpoint pathway and reversing T-cell exhaustion, it enhances the body’s antiviral immune response, thereby decreasing SFTSV replication indirectly (224).

The primary advantages of HTA lie in its high drug resistance barrier and potential broad-spectrum efficacy, while VTA features direct action and rapid onset. Deepening host target discovery, developing synergistic combination strategies between VTA and HTA, and advancing the clinical translation of immunomodulatory therapies will be key directions for future antiviral research and development.

### 4.5 Host metabolism-targeted antiviral therapy

There exists a close interaction between metabolic disorders and viral infections. On one hand, metabolic dysregulation states can exacerbate the severity of SFTSV infection and influence prognosis (212, 225, 226). On the other hand, viral infection may trigger host metabolic dysfunction, establishing a detrimental loop (227–229). Therefore, interventions targeting critical metabolic pathways may offer a novel approach to improving the prognosis of viral infections. Metformin suppresses cellular autophagy via the AMPK-mTOR pathway, thereby reducing SFTSV viremia levels and mortality in patients with hyperglycemia (228). For patients with hypertension, the administration of CCB antihypertensive drugs, particularly nifedipine, helps mitigate disease severity, alleviate clinical symptoms, and reduce the risk of progression to severe SFTS (230). Lovastatin and fenofibrate, as approved lipid-lowering drugs, can inhibit SFTSV replication and hold potential for conversion into antiviral therapeutic drugs (231). Multiple studies indicate that host lipid metabolism is closely associated with viral infection and may function as an effective antiviral target (232, 233). Taurolithocholic acid (TLCA), a secondary bile acid, indirectly suppresses virus-induced ferroptosis by activating the TGR5-PI3K/AKT-SREBP2 signaling pathway, enhancing the expression of fatty acid desaturase 2 (FADS2). *In vitro*, it inhibits SFTSV replication and mitigates host inflammatory responses, thereby protecting mice against fatal infection (227). Supplementing with arginine increases platelet nitric oxide (Plt-NO) concentration, inhibits platelet activation, accelerates platelet recovery, and restores T-cell CD3-ζ chain expression. This promotes viral clearance and reduces liver injury marker (AST/ALT) levels. However, it did not significantly decrease mortality rate sand necessitates additional examination (226).

### 4.6 Exploration of combination drug therapy strategies

Combination therapy strategies demonstrate significant advantages in antiviral treatment by effectively suppressing viral replication through complementary or synergistic mechanisms. Combined use of ribavirin with interferon (IFNα/β/γ) significantly enhances antiviral efficacy and diminishes the necessary therapeutic dosages each drug. Experimental data indicate that this combination regimen at their respective EC_90_ concentrations reduces viral titers by more than three log_10_ levels, whereas monotherapy reduces titers by only about one log_10_ level. Concurrently, the reduced required dosage decreases potential toxicity (234). Drugs that activate innate immune mechanisms, such as the vitamin D derivative alfacalcidol (122) and the immunomodulator tilorone (marketed as Amixin/Lavomax) (235), enhance the antiviral state of host cells, mitigate tissue damage, and lower mortality rates. When such immunomodulators are combined with the direct-acting RNA polymerase inhibitor T-705, their complementary mechanisms produce significant synergistic effects that far exceed the sum of their individual actions. This dual strategy of “immunomodulation plus direct antiviral action” offers a new therapeutic alternative to patients with SFTSV, particularly those with severe disease. Tocilizumab targets cytokine storms by blocking IL-6 receptors and synergistically inhibits core inflammatory pathways with corticosteroids, which possess broad-spectrum anti-inflammatory effects, thereby effectively controlling excessive inflammatory responses. Clinical data from critically ill patients show that, compared to corticosteroid monotherapy (14-day mortality rate of 39.2%), this combination regimen significantly reduces mortality to 11.8%, lowering the risk of death by 79%, and can serve as a treatment option for SFTS (236). Additionally, the combined use of interferon signal enhancers with recombinant interferon can mitigate its associated side effects by reducing the required dose of recombinant interferon needed to achieve optimal therapeutic efficacy. Studies indicate that the combination of kaempferide with low-dose IFN-β can enhance antiviral effects (162).

In summary, combination therapy strategies may broadly encompass the co-administration of virus-targeted and host-targeted drugs, the combination of immunomodulators and direct-acting antivirals, and multi-targeted drug combinations, demonstrating promising application potential. Future research should further explore combination therapy regimens. By combining drugs with different mechanisms of action, synergistic effects can be harnessed to enhance antiviral efficacy, reduce single-agent dosages to minimize cytotoxicity, and delay the emergence of drug resistance. With the application of AI technologies, researchers can extract key molecular features from massive experimental datasets to systematically identify synergistic or antagonistic relationships between drug combinations, significantly accelerating the discovery and development of novel drug combinations (237).

### 4.7 Progress in vaccine development

Research on SFTSV vaccines involves a variety of technical methodologies, such as DNA vaccines, attenuated live vaccines, viral vector vaccines, and mRNA vaccines. A multi-antigen DNA vaccine, designed based on a consensus sequence, has demonstrated the ability to induce strong neutralizing antibody and T cell responses at low doses in both murine and ferrets, resulting in complete protection from lethal SFTSV challenge. However, this approach necessitates the use of electroporation for delivery and requires multiple immunizations (238). Nanopatterning technology significantly enhances the loading capacity and coating efficiency of DNA vaccines by enhancing the hydrophilicity and biocompatibility of microneedles, without compromising DNA stability. *In vivo* studies demonstrate that this technology effectively elicits robust cellular immune responses, particularly from CD8+ T cells, indicating promising applications (239). In contrast, recombinant SFTSV attenuated live vaccines derived from the HB29 strain(genotype D) (rHB29NSsP_102_A and rHB2912aaNSs) demonstrate the ability to induce cross-protection against B/D genotype strains following a single immunization dose, demonstrating favorable safety and genetic stability profiles. However, further evaluation is warranted concerning their potential risks in immunocompromised individuals, due to the theoretical risk of virulence reversion (240). A live attenuated recombinant vesicular stomatitis virus (rVSV)-based vaccine induces high titers of neutralizing antibodies against SFTSV after a single dose, provides cross-protection against HRTV, and demonstrates potential for post-exposure prophylaxis. Its favorable storage stability further increases practical applicability (241, 242). The rVSV vaccine expressing the glycoprotein of the SFTSV AH12 strain (genotype F) shows reduced replication *in vitro* and demonstrates a favorable safety profile in IFNAR^-^/^-^ mouse models, while also inducing high-titer cross-neutralizing antibodies (241). Notably, vaccine derived from the HB29 strain (genotype D) generates a weaker neutralizing antibody response in immunocompetent C57BL/6 mice, indicating that its replication and subsequent immune response may be limited (242). The Ad5 (Adenovirus type 5) vector vaccine induces robust levels of neutralizing antibodies and Th1/Th2 responses through the expression of the Gn protein, achieving complete protection in immunodeficient models. Furthermore, Gn demonstrates superior immunogenicity compared to Gc or their co-expression (243). The bivalent rabies virus vector vaccine conferred highly effective protection against both RABV and SFTSV in mice (244). Furthermore, in a mouse model pre-vaccinated with the vaccinia virus, the m8 GPC and m8 N+GPC vaccines enhanced survival rates against lethal doses of SFTSV infection while mitigating tissue damage. This finding indicates the potential utility of such vaccines (245). Additionally, the self-replicating mRNA vaccine pJHL204225, which expresses SFTSV NP, Gn/Gc, and NS antigens and is constructed using the attenuated Salmonella JOL2500 vector, along with the dual expression system pJHL270/pJHL305226, which expresses SFTSV Gn/Gc epitopes and full-length glycoproteins in both prokaryotic and eukaryotic systems, can elicit balanced Th1/Th2 immune responses without the need for exogenous adjuvants. These vaccines generate high levels of antigen-specific IgG, IgM, and neutralizing antibodies, promote CD4+ and CD8+ T cell proliferation and cytokine secretion, and significantly reduce viral load while alleviating tissue damage in human C-type lectin receptor (hDC-SIGN) transgenic mouse challenge models, demonstrating potential for multi-antigen delivery. It is noteworthy that the type and intensity of immune responses elicited by various antigen combinations can differ significantly (246), and the size of the carrier may also affect antigen delivery efficiency and the rate of immune activation (247).

mRNA vaccines have gained prominence as a platform for vaccine due to their rapid development, favorable safety profile, and feasibility for large-scale production (248). mRNA-LNP vaccines targeting Gn and encapsulated in lipid nanoparticles (LNPs) induced potent humoral and cellular immune responses in mice (249). mRNA vaccines encoding full-length glycoproteins provide long-term protection lasting at least five months and confer cross-protection against other Bandaviruses, such as HRTV and GTV. These vaccines also induce robust Th1-biased cellular immune responses. It is important to note that, due to the current immaturity of large-scale mRNA synthesis technologies, using macromolecular proteins as antigens poses certain challenges during vaccine preparation (250). The multi-epitope mRNA vaccine incorporates conserved epitopes from the Gn, Gc, NP, and NSs proteins and utilizes computational design to achieve broad-spectrum immune coverage. However, predictive models indicate that this mRNA may interact with Toll-like receptor 3 (TLR3), which, while potentially enhancing immune activation, also carries the risk of inducing excessive inflammatory responses. Furthermore, the current findings are predominantly based on computational simulations, and their actual immunogenic effects and safety profiles require experimental validation (251).

In summary, SFTSV vaccine research is progressing rapidly through multiple collaborative technological platforms, demonstrating broad application potential. Beyond vaccine prevention strategies relying on neutralizing antibodies, the cellular immune mechanisms induced by vaccines also play a role in combating viral infections. Studies have reported that even non-envelope protein-specific T cell responses can provide some degree of immune protection (238), but further investigation is needed to elucidate the specific mechanisms involved. Despite significant progress to date, the path to clinical application for SFTSV vaccines remains fraught with challenges. Further optimization of antigen design, enhancement of antibody titers, improvement of vector system safety, validation of vaccine efficacy across multiple animal species, and assessment of vaccine coverage against viral variants are all necessary.

## 5 Discussion

In addition to the limitations of drugs such as ribavirin and favipiravir mentioned above, the practical application of strategies like immunomodulatory therapy and anti-infective treatment in the clinical management of SFTS remains highly controversial and significantly constrained (252, 253). Additionally, the absence of a cohesive disease evaluation system and standardized treatment regimens may expose patients to the risk of overtreatment. Certain drugs may also exacerbate immune dysfunction and elevate the risk of subsequent infections (254, 255).

The development of antiviral drugs for SFTSV encounters numerous obstacles. First, it is challenging to obtain sufficient and representative human patient samples and establish research cohorts. This is chiefly attributable to the sporadic nature of the disease and limitations such as inadequate infrastructure in endemic areas. The scarcity of samples not only risks introducing research bias but also forces the development of prevention and treatment strategies to rely heavily on animal models. Secondly, the stringent biosafety level requirements for SFTSV research restrict its widespread conduct. Third, since SFTS primarily manifests in mountainous and hilly areas with limited medical resources, practical attributes such as drug production costs, storage stability, transportation requirements, and user-friendliness must also be thoroughly considered during the early stages of research and development. Furthermore, the basic research on the pathogenic mechanisms of SFTSV is not deep and thorough, and the research on the pathogenic mechanisms, replication efficiency, and sensitivity to antiviral drugs of different genotypes of viruses are still relatively lacking. This may lead to uncertainty regarding the cross-protective effects of drugs or vaccines designed for one genotype on other genotypes. Additionally, studies examining viral genome variation and differences in pathogenicity among distinct genotypes have been confined to localized regions and are susceptible to interference from host factors. Consequently, it remains challenging to draw definitive conclusions.

Research is actively exploring and refining solutions from multiple angles to address the aforementioned challenges in drug development and clinical application. In the process of new drug development, beyond the aforementioned strategies, fully leveraging natural medicinal resources such as traditional Chinese medicine (195, 223) and plant extracts with antiviral potential (144) can also represent a significant pathway for discovering new candidate compounds. The repurposing of existing pharmaceuticals is particularly well-suited for resource-limited regions due to its advantages of established clinical safety, rapid translation pathways, and the affordability and easy accessibility of some medications. Regarding screening methods, the emergence of novel, safe, and efficient screening platforms has provided an effective pathway for rapidly evaluating antiviral drug candidates. For example, screening platforms based on microgenome systems, particularly the M-fragment platform (98, 202, 256). The advantage resides in enabling the identification of potential active compounds through *in vitro* screening under lower biosafety levels (BSL-1/2), eliminating the need to use infectious live viruses in biosafety level 3 (BSL-3) facilities. Furthermore, fluorescently labeled recombinant viruses constructed using techniques such as reverse genetics are equally critical. These viruses retain the essential characteristics of the parental virus (257, 258) while enabling real-time visualization of the infection process (136, 259–261). They are not only suitable for efficient high-throughput drug screening, but also contribute to elucidating the molecular mechanisms of infection and pathogenesis, thereby facilitating the development of vaccines and antiviral strategies. To comprehensively enhance drug evaluation capabilities, several validation models and technical platforms are also becoming progressively important. Leveraging multi-species animal models for holistic evaluation, utilizing organoid platforms (223, 262) to simulate specific tissue responses, and employing real-time dynamic technologies (263) for precise monitoring of disease progression establish a robust validation platform for *in vivo* drug efficacy and safety assessment. The establishment of prediction and risk stratification models provides effective references for optimizing clinical therapy and advancing personalized medicine. Analysis based on a mortality risk model, including viral load, activated partial thromboplastin time (APTT), monocyte count, altered mental status, and age, suggests that critically ill SFTS patients or those with low AST levels may derive benefit from glucocorticoid therapy. However, early or high-dose administration should be avoided (264). AI technology demonstrates significant value in assisting the design of personalized treatment plans and enhancing treatment precision through its highly efficient data integration and analytical capabilities (214). A dynamic nomogram model constructed using machine learning algorithms integrates four key indicators, including level of consciousness, lactate dehydrogenase (LDH), AST, and age, to effectively predict patients’ short-term prognosis and identify high-risk individuals likely to benefit from IVIG therapy (265). Another predictive model, UNION-SFTS, stratifies mortality risk based on six variables: viral load, APTT, AST, altered mental status, blood urea nitrogen, and age. This model recommends immunoglobulin or corticosteroid therapy only for high-risk or critically ill patients with a predicted mortality risk ≥12.5% (266). The technical models previously discussed are presently undergoing development and validation. A significant number of the screened compounds lack experimental data derived from animal studies. Moreover, current research platforms are generally inadequate for comprehensively simulating the virus’s entire life cycle and its intricate interactions within the host organism in a singular system. These platforms are limited to replicating specific functions and are predominantly utilized for fundamental research purposes. In the process of establishing predictive models, challenges such as data gaps, inconsistent standardization, and inadequate sample representativeness frequently compromise predictive accuracy. Consequently, the algorithms necessitate further optimization and validation.

The expansion of vectors is exacerbating the transmission risk of SFTSV. Warming climates continue to broaden tick habitats and facilitate the spread of *Haemaphysalis longicornis* through parthenogenesis to regions such as Australia and New Zealand (267). Meanwhile, the East Asia-Australia migratory bird flyway serves as a critical conduit for transoceanic transmission (268). It is endemic in Southeast Asia (43), with serological evidence from Kenya suggesting potential spread risks in Africa (269). Collectively, these findings indicate that SFTSV is exhibiting a tendency toward global dissemination.

However, owing to insufficient monitoring of SFTSV beyond East Asia and limited understanding of its transmission routes, its disease burden is considerably underestimated. HRTV cases have been reported in at least 14 states across the United States. The primary vector of the virus, the *Amblyomma americanum*, continues to expand northward due to climate warming, significantly increasing the potential transmission range of the virus. However, due to the lack of systematic serological surveys, insufficient awareness among clinicians, and the absence of commercial diagnostic tests (reliance on limited testing by the CDC), the current surveillance system is experiencing significant underreporting (270). Meanwhile, in Pakistan’s Faisalabad region, the population exposure rate has reached 17.38% (with agricultural workers facing particularly high risks). Nevertheless, due to monitoring efforts being concentrated in urban areas, limited diagnostic capacity for high-risk rural populations, absence of vector surveillance, and insufficient public awareness and health education, these factors collectively present a potential threat to disease control (271). Globally, there is a shortage of specific antiviral drugs, with the research of pharmaceuticals and vaccines predominantly focused in East Asia. Investigations concerning strains beyond East Asia are insufficient (272).

Furthermore, viral mutations can easily be confused with other hemorrhagic fevers, and inadequate testing resources in medically underserved areas and primary care facilities can result in missed or misdiagnoses, delaying treatment (273). Therefore, there is an urgent need to establish rapid and simple identification methods for variant strains and to enhance molecular surveillance of highly lethal strains (35, 274).

To effectively address the challenges posed by SFTSV, coordinated efforts across multiple levels will be required in the future. First, its pathogenic mechanisms must be thoroughly elucidated to actively explore novel therapeutic strategies, such as combination drug therapies and precision-targeted treatments while, strengthening related technologies and validating of existing drugs. Simultaneously, it is imperative to accelerate the development of specific drugs and regionally adapted vaccines through deepened international collaboration and improved virus information sharing. Furthermore, ongoing research into the local transmission ecology and evolutionary patterns of the virus is essential. This includes strengthening source control, real-time surveillance, and diagnostic capabilities; systematically enhancing medical response levels and public risk awareness; and continuously optimizing clinical treatment strategies.
